# Maternal–Infant Iron Deficiency and Neurologic Sequelae

**DOI:** 10.3390/nu17050841

**Published:** 2025-02-28

**Authors:** Barbara T. Felt, Phu V. Tran

**Affiliations:** 1 Department of Pediatrics, University of Michigan, Ann Arbor, MI 48109, USA; 2 Department of Pediatrics, University of Minnesota, Minneapolis, MN 55455, USA; tranx271@umn.edu

**Overview:**

There has been longstanding interest in the prevalence of iron deficiency (ID) and its effects across the life span. Iron deficiency is a common micronutrient deficiency worldwide and the need for iron is ubiquitous across physiologic systems. Having optimal iron status has positive implications for growth, neurodevelopment, immune function and mental health. However, it is important to understand the mechanisms of iron regulation from its uptake and trafficking at the cellular level, to between regional and organ systems in order to determine opportunities to improve outcomes at the individual and population levels through treatment for ID with optimal timing or prevention.

The publications in this special supplement of *Nutrients* provide an overview of our knowledge to date in several areas. Studies investigate the mechanisms whereby ID or the dual burden of more than one deficiency/toxicity affect the iron status of the brain and other organs as well as neurodevelopmental outcomes. Others investigate the impact of ID on associated medical and mental health outcomes and take into account contexts related to resources/income. Knowledge in these areas is still evolving; however, these papers provide updates across the field of ID and guidance for future national and global policies related to this nutritional area. Papers considering basic mechanisms are summarized first, followed by those more closely related to human outcomes at the clinical and population levels.

**Reviews and basic mechanisms:**

The paper, “*Biomarkers of Brain Dysfunction in Perinatal Iron Deficiency*” by R. Rao [contribution 1], the eleventh in the series, affords a comprehensive primer on perinatal ID and the response to iron treatment. It reviews the mechanisms whereby perinatal ID occurs and potential biomarkers are used to indicate brain iron status. The author argues that due to the known negative effects of ID in the perinatal period on neurodevelopment, it is critical for guidelines on ID treatment to shift the focus from normalizing hematologic markers alone to including indices that better account for and optimize brain iron status for the individual. This is an important idea because evidence points to the prioritization of iron to the red blood cells over the brain cells; consequently, the brain would already have been iron deficient for some time prior to the detection of ID using peripheral biomarkers.

The third publication in the series is by Burkhart et al. [contribution 2], entitled “*Normalization of Fetal Cerebral and Hepatic Iron by Parental Iron Therapy to Pregnant Rats with Systemic Iron Deficiency without Anemia*”. This paper reports on the intramuscular administration of ferric derisomaltoside/iron isomaltoside during pregnancy and the effects on brain iron in the offspring. Without iron supplementation, pups were not viable, but offspring viability and brain iron were maintained with the intervention. The study findings support the prioritization of iron to the brain in a preclinical model and could have important implications for human pregnancy where oral iron is poorly absorbed and adherence rates for taking it are poor.

The seventh paper by Cubello et al. [contribution 3], entitled “*Maternal Iron Deficiency and Environmental Lead (Pb) Exposure Alter the Predictive Value of Blood Pb Levels on Brain Pb Burden in the Offspring in a Dietary Mouse Model: An Important Consideration for Cumulative Risk in Development*”, uses a dual-burden model of pre-pregnancy lead exposure followed by pre-pregnancy and pre-and postnatal ID or iron-sufficient diets. Maternal and offspring lead and iron status and the relationship between the two are reported. The authors remind the reader that calcium (and lead) that accumulates during growth for the prospective mother are released during pregnancy to meet the offspring’ s needs for growth, an additional consideration for intragenerational effects of lead exposure. The study demonstrates that ID increased brain lead burden in the offspring and that circulating lead levels alone were not sufficient to estimate the burden of lead in the brain. This work has implications for the multifactorial presentations seen in clinical care.

The first paper in the series by Mukhtarova et al. [contribution 4], “*Influence of Biological Sex and Congenital Iron Deficiency on Neonatal Cytokine Responses*”, extends our knowledge about the role of iron and biological sex on cytokine profile responses. Cord blood mononuclear cell cytokine profiles were studied in response to phytohemagglutinin stimulation of cord blood samples classified as congenital ID according to transferrin saturation < 36% and three or more risk factors. Sex-specific effects of congenital ID were demonstrated on cytokine response profiles. The authors recommend longitudinal investigations of the mechanisms underlying the apparent predisposition by biological sex and the role of fetal ID on immune response to infections and the development of atopic diseases such as asthma later in life.

The ninth paper in the series is by Liu et al. [contribution 5], “*Sex-Specific Effects of Early-Life Iron Deficiency and Prenatal Choline Treatment on Adult Rat Hippocampal Transcriptome*”. This study is elegant in its design and analysis. Long-term changes in hippocampal gene regulation were compared between adult male and female rats that had or did not have prenatal choline treatment and were ID or iron-sufficient during the fetal to early neonatal period. Overall, gene expression findings in the adult hippocampus demonstrated long-term changes, particularly in females who had experienced fetal choline exposure and the adverse event of ID. Given the prevalence of ID and the seemingly great challenge to avoid it, this work may signal methods to rescue outcomes associated with ID.

The 12th paper by Reid and Georgieff [contribution 6], “*The Interaction between Psychological Stress and Iron Status on Early-Life Neurodevelopmental Outcomes*”, reviews the evidence for the dual effects of ID and another common exposure, stress. The paper highlights the intersection between stress and iron homeostasis. Infectious and chronic psychological stress interfere with HPA axis regulation which can in turn affect iron regulation through the activation of hepcidin by cytokine IL-6, a pro-inflammatory agent. Hepcidin works with the iron transport ferroportin to regulate iron absorption, distribution and storage. While both psychological stress and ID have been demonstrated to affect neurodevelopmental outcomes, the impact of both in concert is less explored. The authors advocate for more study into the effects of non-infectious stress on iron regulation, as well as the consequential effects on neurodevelopment.

**Medical and mental health conditions:**

The fifth paper is by Gonzalez-Fernandez et al. [contribution 7], entitled “*Multiple Infections, Nutrient Deficiencies, and Inflammation as Determinants of Anemia and Iron Status during Pregnancy: The MINDI Cohort*”. This interesting publication enrolled pregnant women in Panama to assess whether measures of iron status and factors related to lower middle-income status are associated with the nutritional status, inflammatory biomarkers, and infectious status of the mothers. This study underscores the complexity of evaluating the impact of iron status when multiple factors (environmental, food resources) and their interactions may play a role. The authors caution that, as reported decades ago, treatment for ID anemia should consider the risk of infections, particularly in indigenous populations.

The sixth paper in the series entitled “*Baby Food Pouches, Baby-Led Weaning, and Iron Status in New Zealand Infants: An Observational Study*” by McLean et al. [contribution 8] provides results from the first group to study the effect of baby pouches on iron status and on self-weaning around 6 months of age. The “First Foods New Zealand” observational study found that 23% of enrolled infants in the second half of their first year of life had suboptimal iron status; however, self-led weaning or baby food pouch groups were similar for iron status outcomes. The findings highlight a need for continued monitoring of infants’ iron status during the complementary feeding period.

The paper “*Examining the Double Burden of Underweight, Overweight/Obesity and Iron Deficiency among Young Children in a Canadian Primary Care Setting*” by Borkhoff et al. [contribution 9] is the eighth in the series. This publication discusses the double burden of malnutrition or overweight and other non-communicable diseases (like ID). The authors note that information is available from lower-income-setting studies, but that observations from higher-income settings are few. In a higher-income setting, a larger body mass index was associated with ID in 12–29-month-old infants. The authors suggest that zBMI > 2 should be added to list of important risk factors for ID risk.

The second paper in the series, “*Iron Deficiency and Internalizing Symptoms Among Adolescents in the National Health and Nutrition Examination Survey*” by Fiani et al. [contribution 10], evaluated the National Health And Nutrition Evaluation Survey (NHANES) database over five cycles starting in 2005 for simultaneously available iron measures and mental health ratings. Two methods for defining iron status were assessed. The authors found that in multiracial adolescents, ID with and without anemia was associated with a high risk for depression, and ID with anemia was associated with more severe anxiety. The authors advocate for a larger prospective study on mental health and iron status in teens. This is highly relevant particularly for adolescent females, who are at a higher risk of ID due to the loss of iron through the menstrual cycle.

The 13th publication in this series by Alhammad et al. [contribution 11], “*LRG1 Associates with Iron Deficiency Anemia Markers in Adolescents*” describes the association of leucine-rich -2 glycoprotein1, (LRG1), and biomarkers for ID anemia in children 11–14 years of age. Overall, LRG1 was higher in children with anemia; however, LRG1 was negatively or positively associated depending on the iron status measure. Further studies were encouraged by the investigative team to explore whether LRG1 can be added to the panel of ID markers.

Finally, two studies discussed the association of iron status to medical aspects of sleep. The fourth publication by Ipsiroglu et al. [contribution 12], “Iron Deficiency and Restless Sleep/Wake Behaviors in Neurodevelopmental Disorders and Mental Health Conditions”, seeks to advance the understanding of restlessness related to disorders of sleep initiation and maintenance through a standardized clinical assessment, laboratory studies and interview. The majority of patients had ID, and neurodevelopmental conditions (ADHD and autism), chronic insomnia and sleep-disordered breathing were common. The risk for sleep–wake disorders was increased among those with a family history of ID.

The 10th publication in the series by McWilliams et al. [contribution 13], “*Iron Deficiency and Sleep/Wake Behaviors: A Scoping Review of Clinical Practice Guidelines—How to Overcome the Current Conundrum?*”, discusses the association of ID with common conditions such as restlessness related to sleep and ADHD. The paper reviews guidelines on ID and iron treatment recommendations. The report highlights gaps in data collection and harmonization. In particular, the variability of biomarkers and cutoffs by age among these guidelines is noted, questioning the applicability of the current guidelines. The authors suggest that further work is needed to reach consensus regarding what biomarkers to use for iron status and whether cutoffs should differ by condition. In addition, the authors note the importance of assessing the impact of inflammation on iron biomarker assessments.

